# Long-Distance Dispersal via Ocean Currents Connects Omani Clownfish Populations throughout Entire Species Range

**DOI:** 10.1371/journal.pone.0107610

**Published:** 2014-09-17

**Authors:** Stephen D. Simpson, Hugo B. Harrison, Michel R. Claereboudt, Serge Planes

**Affiliations:** 1 Biosciences, College of Life and Environmental Sciences, University of Exeter, Exeter, United Kingdom; 2 Australian Research Council Centre of Excellence for Coral Reef Studies, James Cook University, Townsville, Queensland, Australia; 3 Department of Marine Science and Fisheries, Sultan Qaboos University, Al-Khod, Oman; 4 Le Centre de Biologie et d’Ecologie Tropicale et Méditerranéenne, l’Université de Perpignan, Perpignan, Pyrénées-Orientales, France; 5 Laboratoire d’Excellence “CORAIL”, Centre de Recherches Insulaires et Observatoire de l’Environnement, Moorea, French Polynesia; Glasgow Caledonian University, United Kingdom

## Abstract

Dispersal is a crucial ecological process, driving population dynamics and defining the structure and persistence of populations. Measuring demographic connectivity between discreet populations remains a long-standing challenge for most marine organisms because it involves tracking the movement of pelagic larvae. Recent studies demonstrate local connectivity of reef fish populations via the dispersal of planktonic larvae, while biogeography indicates some larvae must disperse 100–1000 s kilometres. To date, empirical measures of long-distance dispersal are lacking and the full scale of dispersal is unknown. Here we provide the first measure of long-distance dispersal in a coral reef fish, the Omani clownfish *Amphiprion omanensis*, throughout its entire species range. Using genetic assignment tests we demonstrate bidirectional exchange of first generation migrants, with subsequent social and reproductive integration, between two populations separated by over 400 km. Immigration was 5.4% and 0.7% in each region, suggesting a biased southward exchange, and matched predictions from a physically-coupled dispersal model. This rare opportunity to measure long-distance dispersal demonstrates connectivity of isolated marine populations over distances of 100 s of kilometres and provides a unique insight into the processes of biogeography, speciation and adaptation.

## Introduction

Dispersal drives population dynamics, allows the replenishment of harvested marine species and defines the structure and persistence of marine populations across fragmented and often ephemeral habitat landscapes [1]–[3]. Since most coastal marine organisms are site-attached as adults, connectivity between discreet populations depends on the successful dispersal of planktonic larvae [4]. However, larvae spend days to months developing in the open ocean before settling to new habitats, thus tracking the dispersal trajectories of individuals during the larval stage is not feasible, and the full scale of dispersal remains largely unknown [5], [6]. In recent years, substantial effort has been made to understand patterns of connectivity in marine species between discreet patches of coastal habitat in order to inform conservation efforts [7] and better manage natural resources [8]. At small spatial scales (10 s km), dispersal can facilitate the replenishment of local fished areas by neighbouring protected populations [9], [10]. Over large spatial scales (1000 s km) dispersal can drive the spread of invasive species [11]–[13] and facilitate species range shifts in response to climate change [14]. At intermediate scales (100 s km) dispersal is predicted to allow the recolonisation of disturbed and depleted populations [7], however direct measurements of the successful movement and colonisation of individual larvae at this scale have been thus far elusive.

Many recent studies have focussed on coral reef fish, which are generally highly site-specific to naturally fragmented habitats, thus providing a valuable model system for studying dispersal and connectivity in marine ecosystems. Findings show that recruitment on coral reefs is largely driven by the retention of larvae within their populations of origin [15], [16] combined with immigration from neighbouring populations [9], [17]–[21]. However, the behavioural and ontogenetic characteristics of coral reef fish larvae [4], , broad-scale genetic homogeneity seen in some species [23]–[25] and predictions of coupled-biophysical models [26]–[30] all suggest larvae also have the potential to undertake long migrations during their pelagic phase. Indeed, the vast species ranges seen in many reef fish, some spanning entire ocean basins, suggest that occasional long-distance dispersal or background gene flow along regional stepping-stones must occur to maintain genetic coherence of species and prevent speciation. To date, evidence for long-distance dispersal (100–1000 s km) comes from studies of evolutionary processes that measure historical gene flow [23]–[25], while attempts to infer large-scale patterns of dispersal using oceanographic models [26], [29]–[31] are, as yet, unvalidated estimates of demographic connectivity.

**Figure 1:**
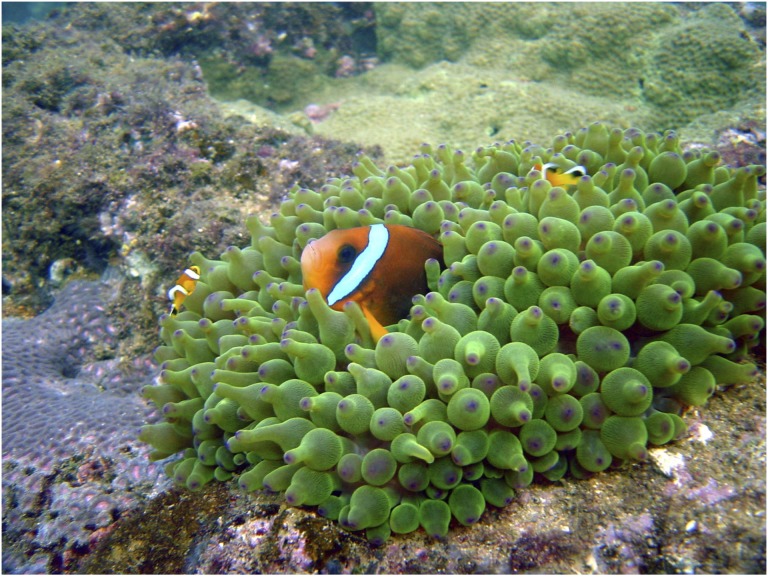
The Omani clownfish, *Amphiprion omanensis,* is endemic to the southern coast of Oman. Adults provide high levels of parental care to their young that hatch with well-developed swimming and sensory capabilities before embarking on a <3 week pelagic larval phase, during which time they may disperse over long distances (>400 km).

Combining direct measurements of successful colonisation following long-distance dispersal with validated estimates of demographic connectivity, we investigate the potential for coral reef fishes to disperse over long distances during only a short pelagic phase. Our study focuses on the Omani clownfish *Amphiprion omanensis* (Fig. 1), which is endemic to the Arabian Sea and found only on shallow coral reef dominated habitat located in two regions of the southern coast of Oman, separated by over 400 km of high exposure sandy shores [32]. This provides a unique opportunity to investigate dispersal between distant populations spanning an entire species range. Over large spatial scales, where gene flow is restricted, genetic assignment tests can be used to identify the origin of individuals provided that discrete populations are genetically distinct and that all populations have been sampled [33]–[35]. Using population-specific assignment thresholds, we determined whether individuals were local-type, first-generation migrants or local-migrant hybrids within each region. We compared our empirical findings to predictions from a simple physically-coupled individual-based dispersal model to determine whether oceanographic current flows could predict long distance dispersal events.

## Materials and Methods

### a) Field sampling of *Amphiprion omanensis* (including Ethics Statement)

During an expedition in December 2006 and January 2007, we sampled 136 clownfish in the northern province of Ash Sharqiyah and 260 in the southern province of Dhofar (Table S1). Fish were collected and released without causing lasting harm and with specific approval for this study from both the University of Edinburgh Ethical Review Committee and the University of Edinburgh Expedition Committee, and with permission from the Ministry of Regional Municipalities, Environment and Water Resources, Sultanate of Oman and Department of Nature Conservation and Wildlife, Governate of Dhofar. A small section of the pectoral fin was removed from each individual and preserved in 95% ethanol. Based on available habitat in each region we estimate that we sampled 5–20% of all individuals in each region.

### b) DNA extraction and microsatellite genotyping

Genomic DNA was extracted from fin tissue samples using standard proteinase K digestion (Gentra Puregene, QIAGEN). Samples were genotyped with a panel of six microsatellite loci previously described for two congeneric species [36], [37] (Table S2) and optimised for *Amphiprion omanensis* in two multiplex reactions. Each locus was sequenced to confirm the presence of simple sequence repeats and integrated to multiplex PCRs on the basis of fragment sizes, heterodimer duplexing and complementarity of melting temperatures. Multiplex PCRs consisted of 1 µl genomic DNA, 0.2 µl MgCl_2_, 1.5 µl dNTPs, 1 µl buffer, 0.5 µl *Taq* polymerase, and 1 µl H_2_O. Multiplex A contained 0.8 µl forward and reverse primers for loci *Ao120*, *Ao84* and *AoCF3*. Multiplex B contained 0.6 µl forward and reverse primers for loci *Ao55* and *Ao22*, and 1.2 µl of each primer for locus *AoCF11*. All PCR reactions were performed on a Eppendorf Mastercycler ep with the following cycling conditions: an initial denaturation of 2 min at 94°C followed by 10 cycles of 45 s at 94°C, 45 s at 60°C (multiplex A) or 66°C (multiplex B), and 45 s at 72°C, followed by 20 cycles of 45 s at 94°C, 45 s at 55°C (multiplex A) or 61°C (multiplex B), and 45 s at 72°C, with a final elongation period of 45 min at 72°C. Amplified PCR products were analysed on a CEQ 8000 Genetic Analysis System (Beckman-Coulter, Fullerton CA) and the resulting electrophoregrams were scored using CEQ 8000 software. Alleles were scored manually (twice, blind) to eliminate scoring errors.

### c) Population genetics statistical analysis

Samples were grouped within northern and southern regions. Nei’s unbiased expected and observed heterozygosity [38], number of alleles and allelic richness over all samples, and Weir & Cockerham’s estimator of inbreeding *F_IS_* [39] were computed in FSTAT v.2.9.3.2 [40]. Observed genotypes were tested for linkage disequilibrium and departures from Hardy-Weinberg equilibrium (HWE) due to heterozygote deficiency at each locus with genotypes randomised among samples as implemented in ARLEQUIN v3.5 [41]. Significance of multiple tests was assessed with sequential Bonferroni corrections applied for multiple comparisons [42]. The occurrence of null alleles and large allele drop-outs were assessed at each locus using MICROCHECKER v.2.2.3 [43]. Analysis of molecular variance (AMOVA) and pairwise genetic distances within and between provinces (*F_ST_*) were computed in ARLEQUIN. Missing data accounted for 0.9% and 1.5% of the data in northern and southern populations, respectively.

### d) Assignment tests and study-specific assignment thresholds

We applied a model-based Bayesian clustering method implemented in structure 2.3.3 [35], using a Markov Chain Monte Carlo (MCMC) resampling procedure, to estimate the most parsimonious allocation of samples to distinct genetic clusters and distinguish between local-type, first-generation migrants and local-migrant hybrids. With the optimum solution (K = 2), we performed 10 independent runs using 100,000 MCMC iterations with a burn-in period of 50,000 steps and computed the arithmetic mean of posterior probabilities of assignment amongst runs.

To determine population-specific thresholds of assignment we first used a conservative threshold of 0.9 [44] to identify northern and southern-type individuals and determine unbiased allelic frequencies for each region. We then randomly selected 100 individuals from each region and simulated 5,000 local-type individuals for each population and 10,000 north-south hybrid individuals using hybridlab v1.0 [45]. By examining the distribution of posterior probabilities of all 20,000 simulated individuals (5 runs with K = 2, 50,000 iterations and burn-in of 50,000) we identified population-specific thresholds of assignment that give a 95% probability of correct allocation of individuals to local-type, first-generation migrant and north-south hybrid classes in our samples (Fig. S1).

### e) Physically-coupled individual-based Lagrangian stochastic dispersal model

We constructed and parameterised an *a priori* dispersal model to simulate dispersal of the larval stage of *A. omanensis*. The model was forced using daily surface current data obtained from the Navy Coastal Ocean Model (US Naval Research Laboratory) for the period 2005–2008, and simulations of larvae released from both northern and southern locations were made for the *A. omanensis* spawning period during three successive seasons (see Information S1). The proportions of larvae that were retained locally, were dispersed to distant reefs or did not settle were retained from each simulation for comparison with our empirical measurements of long-distance dispersal.

## Results

A total of 136 *A. omanensis* individuals were collected in the northern province of Ash Sharqiyah and 260 individuals from the southern province of Dhofar. These two populations are separated by over 400 km of high exposure sandy shore with no suitable habitat and representing the limits of the species range. We found significant genetic differentiation between the two regions (F_ST_ = 0.042; Information S1; Fig. S2), with higher genetic diversity in the southern population (Table S3). Significant departures from HWE due to heterozygote deficiency were observed for three of six loci in both northern and southern regions after Bonferroni correction (Table S3). Locus *Ao84* showed significant departure from HWE in the northern populations only, whereas *AoCF11* showed evidence of heterozygote deficiency in the southern populations. Only *Ao120* showed no significant deviation from HWE expectations, although expected heterozygosity was marginally higher than expected in the southern population. The underlying causes of heterozygote deficiencies were likely due the presence of genetic sub-structure within southern and northern populations that were not captured in our sample. Allelic richness was higher in the southern population, however the mean observed heterozygosity was comparable between regions. Tests for genotypic disequilibrium identified 2 of 30 pairwise comparisons as showing significant linkage after Bonferroni correction (*p*<0.0017).

**Figure 2:**
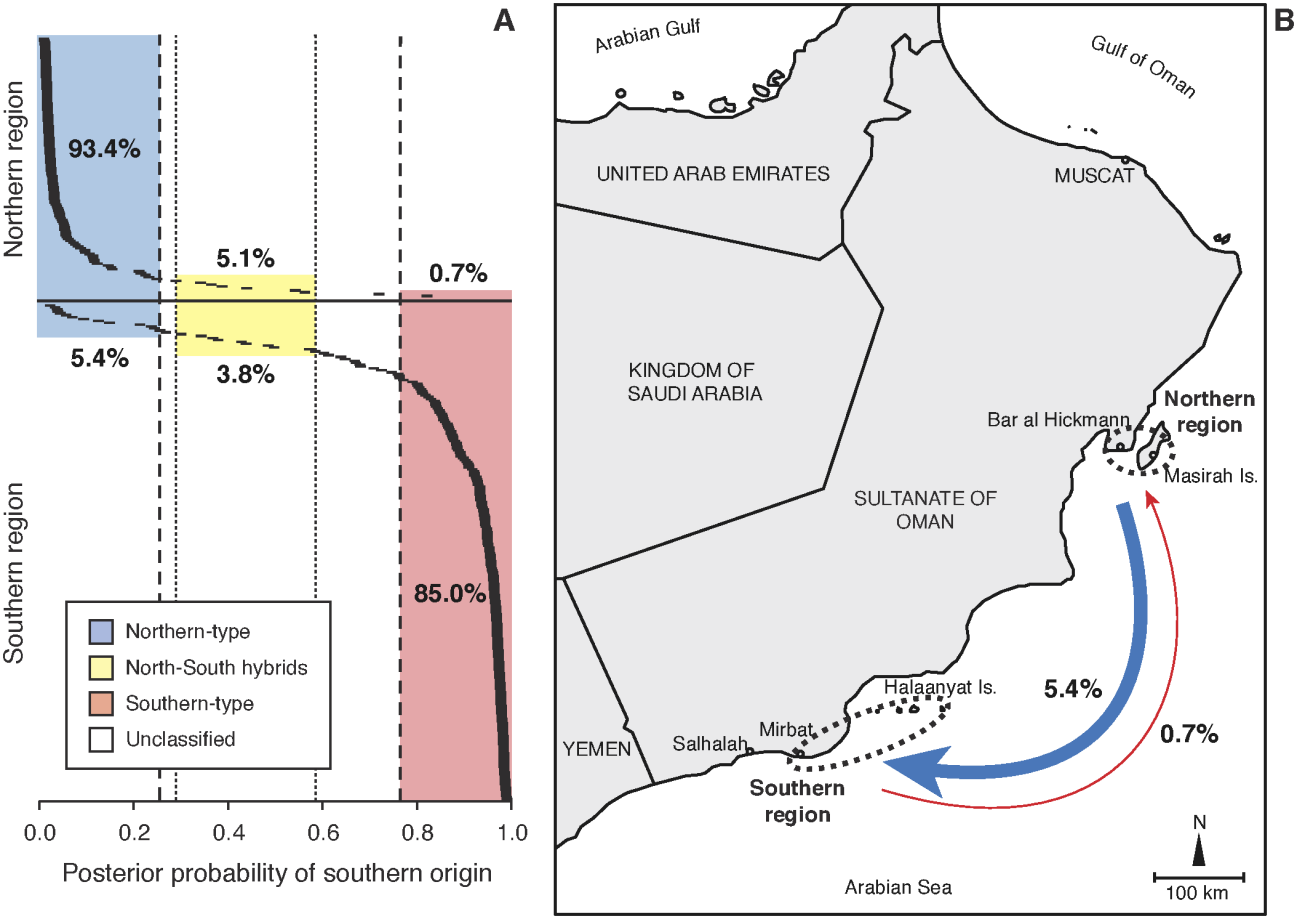
Long-distance dispersal of the Omani clownfish. (a) Bayesian clustering analysis identified individuals that had migrated to distant populations over 400 km from their natal origins. Dashed lines indicate the thresholds of assignment of individuals to northern and southern types. Dotted lines indicate the thresholds of assignment of individuals as north-south hybrids. Percentage values indicate proportion of assigned individuals to different types in each region. (b) The southern Omani coastline has two regions of coral reefs separated by over high exposure sandy shores. The Indian summer monsoon is the main driver of both atmospheric and oceanic regimes causing strong southeasterly winds during summer months (the Khareef monsoon), which then reverse to weaker northeasterly winds during the winter monsoon. These current regimes favour southward dispersal of the Omani clownfish.

Using assignment tests, we detected an asymmetrical dispersal pattern between the two regions with a higher occurrence of southward dispersal than vice versa (Fig. 2a). Our analysis revealed a total of 14 migrants (5.4% of locally sampled individuals) in the southern province of Dhofar that had originated in the northern province of Ash Sharqiyah, but only 1 individual (0.7% of locally sampled individuals) identified as having made the opposite journey (Fig. 2b). Study-specific thresholds were 0.741 and 0.765 for the northern and southern regions respectively, and minimum and maximum thresholds to distinguish hybrid individuals from local-type were 0.413 and 0.711 respectively. Study-specific thresholds were 0.741 and 0.765 for the northern and southern regions respectively, and minimum and maximum thresholds to distinguish hybrid individuals from local-type were 0.413 and 0.711 respectively. Individuals identified as migrants were collected in multiple locations and included both adults and juveniles, demonstrating that some migrating larvae had subsequently become successfully integrated into local populations since maturation in clownfish is socially mediated [36].

In addition to first-generation migrants, we identified 17 north-south hybrids, 10 (3.8% of locally sampled individuals) in the south and 7 (5.1% of locally sampled individuals) in the north. The majority of individuals sampled in each population (221 or 85.0% in the south and 127 or 93.4% in the north) were identified as local-type (Fig. 2a). An additional 15 individuals in the south and 1 in the north could not be confidently distinguished from local and local-migrant hybrid types based on population-specific modelled assignment thresholds. The greater number of unclassified individuals in the southern population is most likely due to the limited proportion of individuals sampled (we estimate 5–20%), greater genetic diversity and higher immigration rates in this population pertaining to a less distinct genetic signature than the northern population and a more conservative minimum threshold of assignment. While migrants and local-migrant hybrids are minority components of the overall population, this study highlights the dispersal potential of pelagic larvae and demonstrates that exchange of larvae between distant populations is likely to be a regular background process rather than one restricted to rare and stochastic events.

The measured southward bias of dispersal matched *a priori* predictions of a physically-coupled individual-based dispersal model parameterised for the larval stage of *A. omanensis* and forced using seasonal current data. Movement of larvae released into the model between December to February, when *A. omanensis* usually reproduces, was predominantly southward as a result of the winter monsoon (Table S4). Conversely, realisations of the model outside of the clownfish spawning season (March/April) predicted northward movement of larvae due to the onset of the summer Khareef monsoon. Depending on the date (between 1 December to 30 January) and year of release, a small percentage (ranging from 0.00 to 5.10%) of larvae were exchanged between the northern and southern populations within a single month and ‘settled’ to appropriate habitat within the 40 days competency period. Averaged over the three winter spawning seasons, the transport of larvae from the northern region to southern reefs was 0.06% to Mirbat and 0.32% to the Halaaniyat Islands, while northward exchange was ≤0.01% (Table S4). Although quantitatively sensitive to initial population size and associated output of larvae, which is not known, qualitatively the model consistently predicted higher migration rates from the northern to the southern populations as seen in our direct measures of long-distance dispersal. Our combined empirical and modelling results for *A. omanensis* suggest that regional current regimes directly influence the dispersal of larvae between southern and northern extremes of its species range.

## Discussion

Geography, oceanography, ecology and behaviour combined favourably in this study system to provide a unique opportunity to empirically measure connectivity between distant populations of a coral reef fish. We found demographically relevant bidirectional exchange of *A. omnanensis* individuals between two regions of suitable coral reef habitat separated by >400 km of high exposure sandy shore, representing the longest direct measure of larval dispersal for any marine fish species to date. Local immigration rates were 5.4% in the south and 0.7% in the north, indicating a biased southward exchange, and these rates matched *a priori* predictions from a physically-coupled individual-based dispersal model (maximum connectivity in a single spawning event 5.1% and 0.1% respectively), which simulated passive larval transport in realistic current fields during the reproductive season. Local migrants were found at all life stages suggesting that these long-distance dispersal events occurred over multiple events and are a regular phenomenon rather than a process limited to chance [46]. Additionally, we identified individuals with mixed north-south genotypes in both regions demonstrating social and reproductive integration of migrants into local breeding populations.

The combination of rarity and conspicuousness meant that our team of 22 divers over 92 dives sampled an estimated 5–20% of all *A. omnanensis* individuals, providing subsequent analyses with sufficient discriminatory power. With more markers and a higher proportion of individuals sampled we would expect intra-regional patterns in dispersal and retention to also become evident as in previous local-scale studies of congeneric species [15], [16], [19]–[21], [36], and would allow the 16 individuals that could not be distinguished between local and local-migrant hybrid types to be confidently assigned. Nonetheless our approach, based on qualifying the unique genetic signature of regional populations, provides a powerful tool to identify local migrants and measure connectivity in marine organisms that is applicable to a wide range of species.

Harvested and threatened populations under spatially variable levels of exploitation, or exposed to environmental stressors including fluctuations in habitat quality and climate change, are offered substantial resilience if replenishment from far beyond the range of local impact is possible. While there are obvious benefits to staying close to home [16], the spatially heterogeneous landscape of coral reef habitats may further confer selective advantage to long-distance dispersers that carry novel mutations across vast stretches of open water. Theory suggests that low rates of migration can rescue individual populations from local extinction and ensure long-term prevalence of species [47]–[48], although until now the nature of long-distance larval exchange in coral reef fishes has remained elusive. Our finding of regular exchange between distant populations explains how seemingly isolated populations maintain their genetic coherence, preventing local adaptation-driven ecological separation that leads to reproductive incompatibility and ultimately speciation. Additional studies using the same geographic case study but comparing a range of contrasting species would enable the influences of life history and oceanography on long-distance dispersal to be determined.

The persistence of spatially structured populations is a factor of both local growth rates and connectivity between populations [49], [50]. Coral reef associated organisms are naturally fragmented and dispersal amongst patches is a fundamental aspect of population dynamics. For the Omani clownfish, the persistence of populations in the northern and southern provinces of the Arabian Sea appears heavily dependent on larval connectivity and larval retention within each province. However, even the low levels of immigration observed between provinces have importance consequences for demographic processes. The greater immigration from north to south indicate a shortfall in local retention in the southern population [49], [50] and a dependence on immigration from the northern population, which itself is comparatively more isolated. In the context of marine spatial planning, these asymmetries in connectivity patterns would warrant a need for greater protection of the northern population. If such high levels of demographic connectivity can be expected between two populations separated by over 400 km, populations in continuous reef habitats or complex barrier reef systems are likely to be much more homogeneous than previously assumed.

Our study also demonstrates that simple physically-forced models can give valuable predictions for realised patterns of connectivity. It is likely that if ocean currents are important for driving the long-distance dispersal events we observed, for fishes with prolonged pelagic larval durations (weeks to months), including many commercially important coastal fishes, long-distance dispersal may be even more prevalent [27], [51], [52]. Further development of the model would allow an ensemble of parameter sets, capturing temporal variability in circulation and the full range of phenological and larval biological traits to be used to generate distributions of self-recruitment and long-distance dispersal for other species in the region. The ability to predict the degree of connectivity between fragmented populations will allow fisheries and conservation managers and marine spatial planners charged with developing national and regional networks of marine protected areas to better manage these populations [53], [54]. Furthermore, better characterisation of the tails of dispersal kernels would provide valuable insight into the ecological and evolutionary processes of biogeography, speciation and potential for species to adapt to current and future climate change.

## Supporting Information

Figure S1 **Frequency distributions of simulated genotypes for the derivation of population-specific assignment thresholds.** A total of 5,000 northern-type, 5,000 southern-type and 10,000 north-south hybrid genotypes were simulated using location specific allelic frequencies. Bayesian clustering analysis determined the posterior probability of assignment of each simulated individual to either northern or southern populations.
(TIFF)

Figure S2 **Scatterplot of the two main components of the discriminant analysis of principal components in four populations of the Omani clownfish** ***Amphiprion omanensis.*** Sampled populations are shown using different colours and 95% inertia ellipses and dots represent each individual in the sample. The x-axis represents 82.9% and the y-axis represents 9.6% of genetic information retained in each discriminant function (inset).
(TIFF)

Table S1 **Sampling sites and number of samples collected.**
(PDF)

Table S2 **Details of six polymorphic dinucleotide microsatellite loci developed for** ***Amphiprion omanensis*** **.**
(PDF)

Table S3 **Details of genetic analysis of each marker.** Number of alleles (*Na*), observed heterozygosity (*Ho*), expected heterozygosity (*He*), the inbreeding coefficient (*F*), and departure from Hardy-Weinberg’s equilibrium (HWE) were calculated for each locus.
(PDF)

Table S4 **Connectivity matrices (% of larvae successfully reaching a reef) for simulated dispersal events.** The upper three panes are yearly averages (10 realisations); the lower panes are overall means, maximum values, standard deviation, coefficient of variation of connectivity and number of non-zero larval transport events over all 30 realisations. Matrices read from-row to-column; grey cells indicate long-distance dispersal. Mirbat and Halaaniyat combine to form the southern region; Masirah and Bar Al Hickmann (MasBAH) combine to form the northern region (see Fig. 2B).
(PDF)

Information S1 **Physically-coupled individual-based Lagrangian stochastic dispersal model; Graphical representation of genetic variation in the Omani clownfish; Supplementary References.**
(DOCX)
