# Supplementary figures and images for: Long-Distance Dispersal via Ocean Currents Connects Omani Clownfish Populations throughout Entire Species Range
Source: PLoS One. 2014 Sep 17;9(9):e107610. doi: 10.1371/journal.pone.0107610 (PMC4167857; doi:10.1371/journal.pone.0107610)

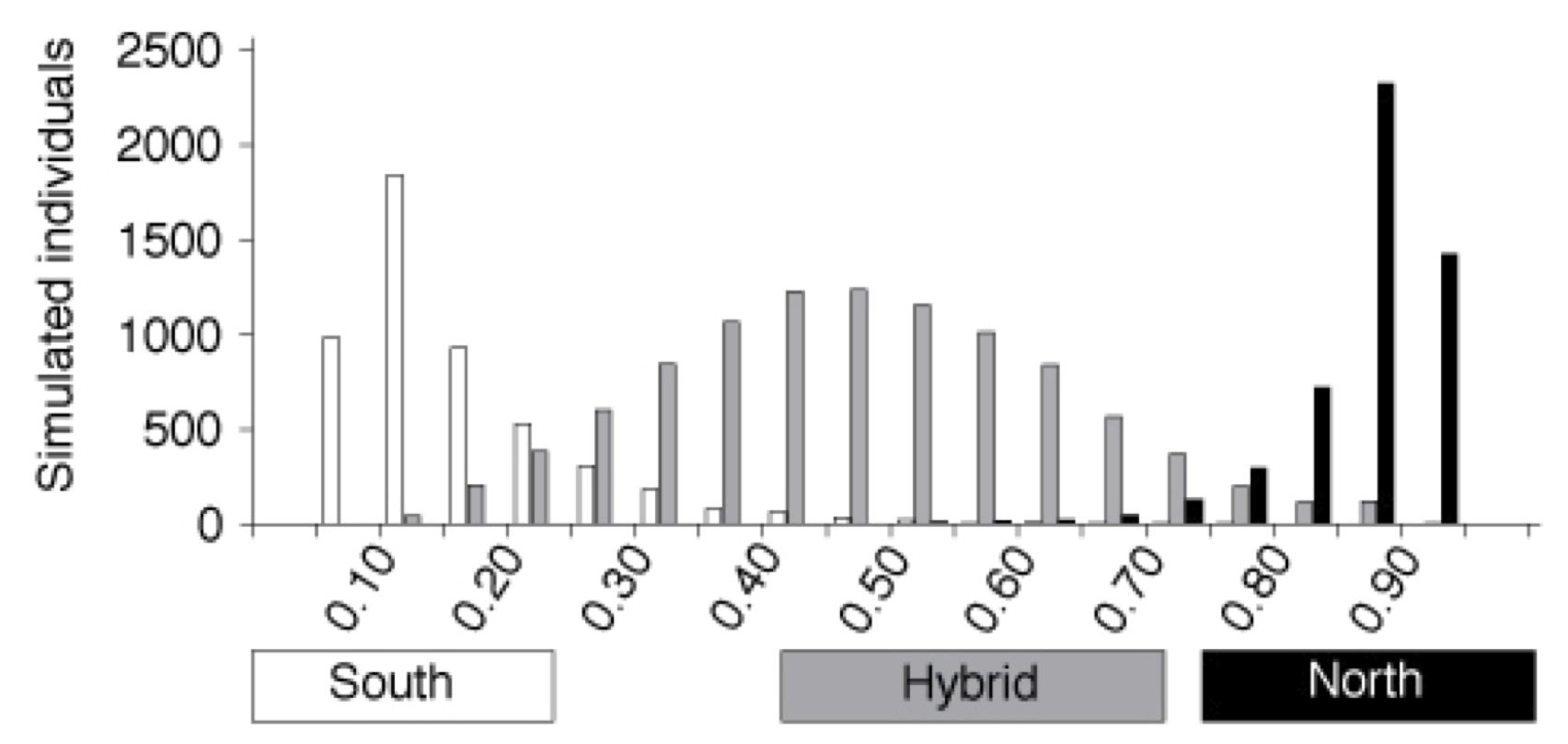

Supplement: Figure S1 — Frequency distributions of simulated genotypes for the derivation of population-specific assignment thresholds. A total of 5,000 northern-type, 5,000 southern-type and 10,000 north-south hybrid genotypes were simulated using location specific allelic frequencies. Bayesian clustering analysis determined the posterior probability of assignment of each simulated individual to either northern or southern populations. (TIFF) [file pone.0107610.s001.tiff]

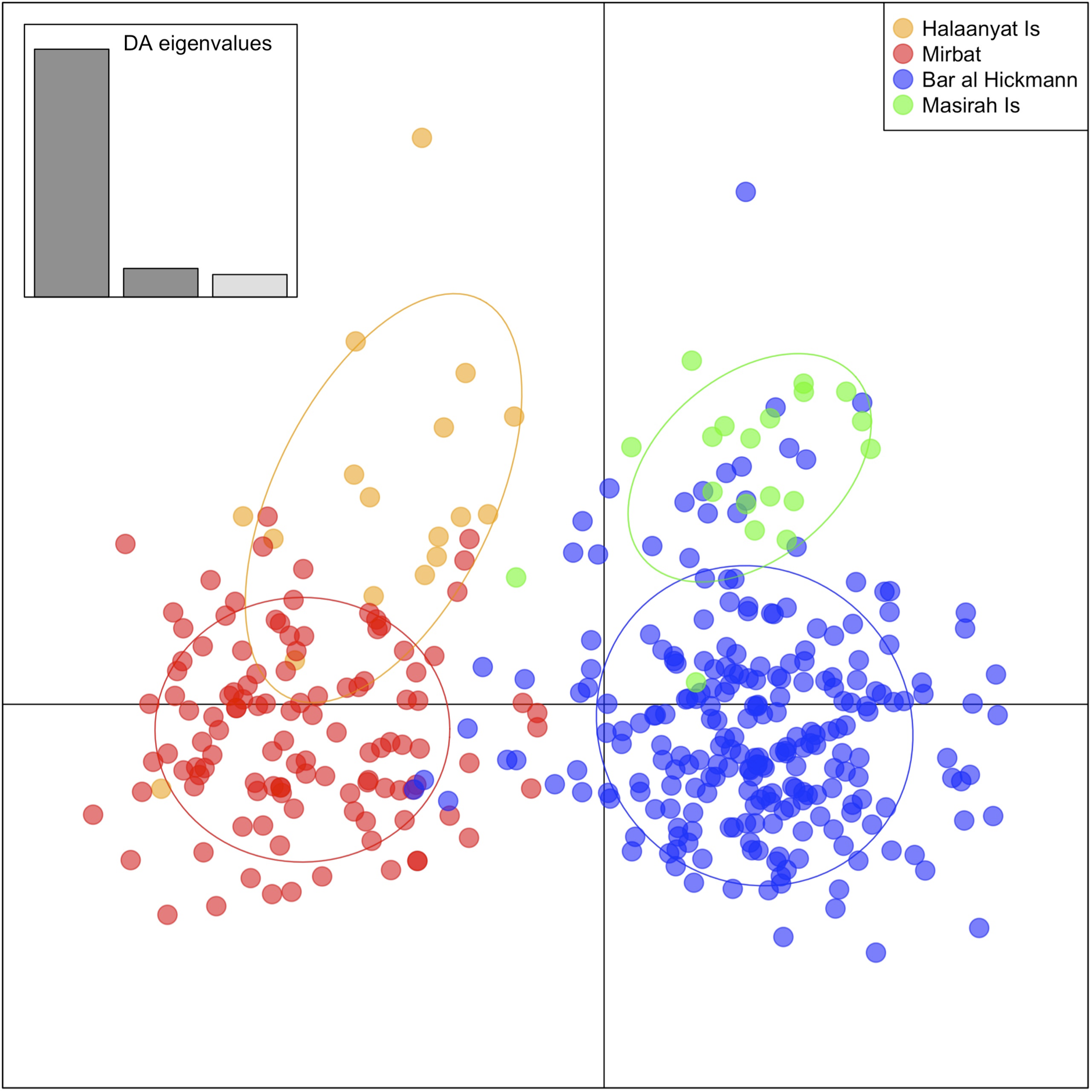

Supplement: Figure S2 — Scatterplot of the two main components of the discriminant analysis of principal components in four populations of the Omani clownfish Amphiprion omanensis. Sampled populations are shown using different colours and 95% inertia ellipses and dots represent each individual in the sample. The x-axis represents 82.9% and the y-axis represents 9.6% of genetic information retained in each discriminant function (inset). (TIFF) [file pone.0107610.s002.tiff]
