# Supplementary material for: Long-Distance Dispersal via Ocean Currents Connects Omani Clownfish Populations throughout Entire Species Range
Source: PLoS One. 2014 Sep 17;9(9):e107610. doi: 10.1371/journal.pone.0107610 (PMC4167857; doi:10.1371/journal.pone.0107610)
Supplement: Table S1 — Sampling sites and number of samples collected. (PDF) [file pone.0107610.s003.pdf]

	Region (Province)	Site	Location	No. samples
**Northern Population**	**Bar Al Hickmann** (Ash Sharqiyah)	BAHW2	20°23'40.57"N 58°12'35.12"E	95
		BAHW2.5	20°25'7.45"N 58°11'36.62"E	3
		BAHW3	20°25'40.65"N 58°11'43.04"E	2
		BAHE1	20°21'57.37"N 58°27'39.71"E	1
		BAHE2	20°21'44.63"N 58°27'33.68"E	2
				**17**
	**Masirah Island** (Ash Sharqiyah)	MIS0	20°10'4.22"N 58°39'31.94"E	32
		MIS1	20°9'52.86"N 58°38'0.96"E	48
		MIS2	20°11'19.74"N 58°37'35.70"E	37
		MIS3	20°12'36.66"N 58°37'30.84"E	2
				**119**
**Southern Population**	**Mirbat** (Dhofar)	Heno Bay	16°57'33.54"N 54°45'21.11"E	44
		Hoons Bay	16°56'36.19"N 54°48'22.15"E	45
		Alto Bay	16°57'4.69"N 54°49'10.81"E	29
		Reccys Bay S	16°57'28.36"N 54°49'40.14"E	22
		Reccys Bay N	16°57'35.19"N 54°49'39.64"E	13
		The Spit	16°57'27.97"N 54°50'23.78"E	10
		Flip Flop Bay	16°57'41.96"N 54°50'55.15"E	10
		Witches Bay W	16°57'56.90"N 54°52'56.00"E	11
		Witches Bay E	16°57'56.61"N 54°53'0.27"E	10
		Cable Bay	16°57'56.28"N 54°53'20.49"E	38
		Wadi Bayt Said E	16°59'16.98"N 54°57'36.93"E	9
				**241**
	**Jazir Al Halaaniyat** (Dhofar)	As Sawdah E	17°28'48.75"N 55°52'39.39"E	1
		As Sawdah West Bay	17°29'8.40"N 55°49'55.01"E	2
		Al Halaaniyah Best	17°29'5.98"N 55°59'21.84"E	3
		Al Halaaniyah Island	17°28'53.51"N 56° 2'30.58"E	3
		Al Halaaniyah SE	17°30'9.43"N 56° 5'21.27"E	4
		Al Halaaniyah ST2	17°29'6.36"N 55°59'35.04"E	4
		Al Halaaniyah SW Bay	17°29'24.19"N 55°58'26.28"E	2
				**19**
			**Grand Total**	**396**
