# Supplementary material for: Long-Distance Dispersal via Ocean Currents Connects Omani Clownfish Populations throughout Entire Species Range
Source: PLoS One. 2014 Sep 17;9(9):e107610. doi: 10.1371/journal.pone.0107610 (PMC4167857; doi:10.1371/journal.pone.0107610)
Supplement: Table S2 — Details of six polymorphic dinucleotide microsatellite loci developed for Amphiprion omanensis . (PDF) [file pone.0107610.s004.pdf]

Locus	Primer sequences (5'-3')	Core repeat	Size range (bp)	No. alleles
Ao120	F: ACC CCA ACA GGT TCC ATT TC<br>R: GAC GGC CTC GAT CTG CAA GCT GA	$(AC)_9$+$(CA)_9$	430-456	16
Ao84	F: AAG GGC CAG TCC TGT CAA ACC CT<br>R: CTG TGA GAG CTC CCG TGT GTA AT	$(TC)_{16}$+$(TG)_{13}$+$(CA)_{21}$	361-381	14
AoCF3	F: GTT CAG CCC TGT ATG ACA TT<br>R: TGC TCT CAT TCC TCT AGT CC	$(CA)_{15}$	259-273	11
Ao55	F: TTA ACT TCC ACA CCC AGT CT<br>R: ACG CTG TGA GAG TCC ATT AT	$(GT)_{30}$	440-548	39
Ao22	F: TCT GTT CAC TTT ATC CAA CTT GT<br>R: GGA GAC ACC AGG CCA ATA GA	$(GT)_{50}$	332-410	44
AoCF11	F: GCT GGT TAC AAC ACC TTG<br>R: GTA ATT GCT GCA AGA CAG	$(TG)_{11}$+$(CA)_{14}$	155-195	19
