# Supplementary material for: Long-Distance Dispersal via Ocean Currents Connects Omani Clownfish Populations throughout Entire Species Range
Source: PLoS One. 2014 Sep 17;9(9):e107610. doi: 10.1371/journal.pone.0107610 (PMC4167857; doi:10.1371/journal.pone.0107610)
Supplement: Table S3 — Details of genetic analysis of each marker. Number of alleles (Na), observed heterozygosity (Ho), expected heterozygosity (He), the inbreeding coefficient (F), and departure from Hardy-Weinberg’s equilibrium (HWE) were calculated for each locus. (PDF) [file pone.0107610.s005.pdf]

Locus	*Ao120*	*Ao84*	*AoCF3*	*Ao55*	*Ao22*	*AoCF11*
Northern region (N = 136)						
Na	8	10	5	25	24	14
Ho	0.853	0.838	0.088	0.765	0.750	0.727
He	0.822	0.769	0.164	0.922	0.923	0.794
F	0.858	0.979	0.004	0.004	0.004	0.033
HWE *P*-value	0.171	< 0.001	< 0.001	< 0.001	< 0.001	0.009
Southern region (N = 260)						
Na	16	14	10	39	43	17
H_O	0.770	0.833	0.336	0.780	0.702	0.591
H_E	0.846	0.892	0.560	0.951	0.953	0.757
F_{IS}	0.004	0.013	0.004	0.004	0.004	0.004
HWE *P*-value	0.044	0.029	< 0.001	< 0.001	< 0.001	< 0.001

Adjusted P-value for F_{IS}: 0.00417
