# Supplementary material for: Long-Distance Dispersal via Ocean Currents Connects Omani Clownfish Populations throughout Entire Species Range
Source: PLoS One. 2014 Sep 17;9(9):e107610. doi: 10.1371/journal.pone.0107610 (PMC4167857; doi:10.1371/journal.pone.0107610)
Supplement: Table S4 — Connectivity matrices (% of larvae successfully reaching a reef) for simulated dispersal events. The upper three panes are yearly averages (10 realisations); the lower panes are overall means, maximum values, standard deviation, coefficient of variation of connectivity and number of non-zero larval transport events over all 30 realisations. Matrices read from-row to-column; grey cells indicate long-distance dispersal. Mirbat and Halaaniyat combine to form the southern region; Masirah and Bar Al Hickmann (MasBAH) combine to form the northern region (see Fig. 2B). (PDF) [file pone.0107610.s006.pdf]

	From/to	Mirbat	Halaaniyat	MasBAH
2005-6	Mirbat	7.05	4.72	**0.00**
	Halaaniyat	20.52	10.76	**0.00**
	MasBAH	**0.02**	**0.42**	37.84
2006-7	From/to	Mirbat	Halaaniyat	MasBAH
	Mirbat	7.81	6.57	**0.01**
	Halaaniyat	2.28	2.51	**0.01**
	MasBAH	**0.06**	**0.08**	30.81
2007-8	From/to	Mirbat	Halaaniyat	MasBAH
	Mirbat	0.10	0.00	**0.00**
	Halaaniyat	6.43	14.81	**0.02**
	MasBAH	**0.10**	**0.46**	28.46
Means of 3 years	From/to	Mirbat	Halaaniyat	MasBAH
	Mirbat	4.75	3.61	**0.00**
	Halaaniyat	9.27	9.50	**0.01**
	MasBAH	**0.06**	**0.32**	32.06
Max. exchange	From/to	Mirbat	Halaaniyat	MasBAH
	Mirbat	43.05	28.90	**0.05**
	Halaaniyat	79.00	48.30	**0.10**
	MasBAH	**1.10**	**5.10**	89.70
Std. deviation	From/to	Mirbat	Halaaniyat	MasBAH
	Mirbat	8.67	7.31	**0.01**
	Halaaniyat	15.13	14.06	**0.03**
	MasBAH	**0.03**	**0.22**	1.03
Coefficient Var.	From/to	Mirbat	Halaaniyat	MasBAH
	Mirbat	20.15	25.31	**17.95**
	Halaaniyat	19.15	29.11	**30.00**
	MasBAH	**2.73**	**4.28**	1.15
No. of events	From/to	Mirbat	Halaaniyat	MasBAH
	Mirbat	23	15	**1**
	Halaaniyat	22	23	**3**
	MasBAH	**3**	**4**	30
