# Supplementary material for: Long-Distance Dispersal via Ocean Currents Connects Omani Clownfish Populations throughout Entire Species Range
Source: PLoS One. 2014 Sep 17;9(9):e107610. doi: 10.1371/journal.pone.0107610 (PMC4167857; doi:10.1371/journal.pone.0107610)
Supplement: Information S1 — Physically-coupled individual-based Lagrangian stochastic dispersal model; Graphical representation of genetic variation in the Omani clownfish; Supplementary References. (DOCX) [file pone.0107610.s007.docx]

**Supporting Information**

**Long-distance dispersal via ocean currents connects reef fish populations throughout entire species range**

Stephen D. Simpson, Hugo B. Harrison, Michel R. Claereboudt, Serge Planes

**Physically-coupled individual-based Lagrangian stochastic dispersal model**

***a) Study region*:** The model was developed for the Northern Arabian Sea and the Sea of Oman (15-26ºN 51-65ºE, Fig. 2). Geologically, the northern Arabian Sea forms a relatively deep basin partially isolated from the Indian Ocean by rising chains of seamounts. The shelf is relatively narrow throughout most of the region with the exception of the north and west of the Sea of Oman. The oceanography of the region is dominated by a seasonal switch between a northeasterly coastal current (the Finlatter Jet) driven by the SW summer monsoon (the Khareef, from April-September), which generates a coastal upwelling along the Arabian Sea coast of Oman, and a southwesterly current driven by the NE winds during the winter monsoon (November to March) [51]. This general circulation pattern is altered by numerous meso-scale eddies, some persisting over long periods of time [52] with potentially long-term ecological consequences. It was thus important to encompass this variability in the model through (a) a protracted 8 week period covering the *A. omanensis* spawning season (December-January) and (b) a series of replicate spawning events over 3 consecutive winters. Although actual spawning has not been reported in *A. omanensis*, the observations of adults guarding eggs in early January and the presence of newly settled recruits in January and young juveniles in May combine to indicate a reproductive season within the period of simulated dispersal.

***b) Larval dispersal:*** To evaluate the extent to which larvae disperse under realistic oceanographic conditions we developed a particle-tracking dispersal model (Lagrangian stochastic model, LSM) with 2-hour time steps forced using 1/8º NCOM daily surface current circulation data obtained from the US Naval Research Laboratory (http://www7320.nrlssc.navy.mil/global_ncom/nind.html) [53].

Individual particle (larvae) velocity was calculated for each day as:

*u_p_* = *U_a_*(*x*, *y*) + *u_r_*(*x*, *y*)

where *U_a_* is the NCOM horizontal ocean surface velocity and *u_r_* is a random component added to the horizontal velocity vector using:

*u_r_* = *R*(2*K_h_*/Δ*t*)^1/2^

where *R* is a evenly distributed random deviate between -1 and +1 and *K_h_* is the imposed explicit Lagrangian horizontal diffusion of the form:

*K_h_* = *ε*^1/3^ *l^4/3^*

where *l* is the unresolved sub-grid scale of the flow model (≈25 km) and ε = 10^-9^m^-2^s^-3^ is the turbulent dissipation rate [54].

Larval movement was modelled assuming that larvae drifted passively in the upper layer of the ocean after hatching, and settlement was modelled whereby larvae developed during a pre-competence period of 16 days after which they settled if they encountered a coral reef during a period of maximum competence that lasted for 5 days. After this period of competency, larvae progressively lost their ability to settle up to a maximum pelagic larval duration (PLD) set at 40 days. These parameters were chosen based on published studies of congereric species [15–17,20–21,36], and the modelling was conducted prior to the results of the molecular analyses being known. Settlement behaviour was parameterised as a daily probability of successful settlement and modelled according to the following equation:

*p_s_* = (1 + e*^(Tc^* ^-^ *^age^*^)^)^-1^ (1 + e*^(age - Tk^*^)^ *^β^*)^-1^

where *T_c_* is the duration of the pre-competence period (days), *T_k_* (days) is the end of the competence period (period after which the settlement probability *p_s_* starts to decline) and *β* is a shape coefficient of the settlement function (0.2 in all simulations) which alters the rate at which larvae lose competency after the competence period. When a larva was able to settle, its location was recorded and it was then removed from the pool of drifting pelagic larvae.

Land boundaries were considered reflective and coral habitats suitable for settlement were parameterised as a series of 3 closed polygons covering the actual coral reef areas of the region with a 20 km buffer zone extending offshore around the coral communities. There were no corrections for mortality or predation introduced in the model. Successful recruitment in this model was thus driven solely by transport and settlement and did not include any peri- or post-settlement processes (competition, growth, mortalities).

***c) Simulation trials:*** Ten spawning events (of 2000 particles each) were modelled based on current fields between December 1 and January 30 for each of 3 years (2005-6, 2006-7, 2007-8) to incorporate inter-annual variability, with larvae released from both northern and southern locations. Successful recruitment was assumed for larvae settling in a suitable habitat during the duration (40 days) of each spawning event. The proportions of larvae that were retained locally, that were dispersed over long distances or that did not settle were retained from each simulation for comparison with our empirical measurements of long-distance dispersal. Local retention was defined as settlement of larvae within their own spawning region and long-distance dispersal (subsidy recruitment) was defined as settlement of larvae to areas outside of the region of release [26]. A connectivity matrix was thus built for each of the 30 individual events (3 years x 10 dates) and summary matrices were calculated for each year.

**Graphical representation of genetic variation in the Omani clownfish**

Population differentiation was investigated graphically (Figure S2) using the *adegenet* package [55]. Discriminant analysis of principal components (DAPC; [56]) first employs a preliminary data transformation step using principal component analysis (PCA) to create uncorrelated variables that summarize total variability (e.g., within and between populations). This discriminant analysis maximises between-group variability and provides the best discrimination of multilocus genotypes into predefined clusters.

**Supplementary References**

51 Burkill PH (1999) ARABESQUE: An overview. Deep-Sea Res Pt II 46: 529-547. (DOI 10.1016/s0967-0645(98)00116-7)

52 Tang DL, Kawamura H, Luis AJ (2002) Short-term variability of phytoplankton blooms associated with a cold eddy in the northwestern Arabian Sea. Remote Sens Environ 81: 82-89. (DOI 10.1016/S0034-4257(01)00334-0)

53 Barron CN, Kara AB, Rhodes RC, Rowley C, Smedstad LF (2006) Validation Test Report for the 1/8 Global Navy Coastal Ocean Model Nowcast/Forecast System. Naval Research Laboratory Report NRL/MR/7320--07-9019.

54 Peliz A, Marchesiello P, Dubert J, Marta-Almeida M, Roy C, et al. A study of crab larvae dispersal on the Western Iberian Shelf: Physical processes. J Marine Syst 68: 215-236. (DOI 10.1016/j.jmarsys.2006.11.007)

55 Jombart T (2008) *adegenet*: a R package for the multivariate analysis of genetic markers. Bioinformatics, 24: 1403-1405. (DOI 10.1093/bioinformatics/btn129)

56 Jombart T, Devillard S, Balloux F (2010) Discriminant analysis of principal components: a new method for the analysis of genetically structured populations. *BMC Genetics*, 11: 94. (DOI 10.1186/1471-2156-11-94)
